# DTH: a nonparametric test for homogeneity of multivariate dispersions

**DOI:** 10.1093/bioinformatics/btag178

**Published:** 2026-04-10

**Authors:** Asmita Roy, Jiuyao Lu, Glen A Satten, Ni Zhao

**Affiliations:** Department of Biostatistics, Johns Hopkins University, Baltimore, Maryland, 21205, United States; The Wharton School of Business, University of Pennsylvania, Philadelphia, Pennsylvania, 19104, United States; Department of Gynecology and Obstetrics, Emory University School of Medicine, Atlanta, Georgia, 30307, United States; Department of Biostatistics, Johns Hopkins University, Baltimore, Maryland, 21205, United States

## Abstract

**Motivation:**

Testing for differences in within-group dispersion is a fundamental problem in multivariate data analysis, with direct implications for interpreting group structure and validating statistical assumptions of other analysis such as ANOVA. Existing methods typically construct test statistics either based on the distance of each observation from the group center or on the mean of pairwise dissimilarities among observations within a group. Both approaches can fail when the mean within-group distance is similar across groups but the distributions of the within-group distances differ. This issue is particularly relevant in high-dimensional microbiome data, where outliers and overdispersion can distort the performance of mean-dissimilarity-based tests.

**Results:**

We introduce the non-parametric Distance-based Test for Homogeneity (DTH), which measures dispersion of a group by computing within-group dissimilarity. Difference in dispersion across groups is tested by comparing the distributions of the within-group dissimilarity across different groups. A combination of Kolmogorov-Smirnov and Wasserstein distances are used to construct the difference between the distributions. For more than two groups, pairwise group tests are combined using a permutation-based p-value. Through simulations, we show that our method has higher power than existing tests for homogeneity in certain situations and comparable power in others. For continuous covariates, we offer an heuristic extension of DTH that showed good performance in simulations.

**Availability and implementation:**

The DTH package, along with the code for reproducing all simulations, analyses, and an accompanying vignette, is available at https://github.com/asmita112358/DTH.

## 1 Introduction

One approach to high-dimensional data analysis is to reduce the data to a matrix of pairwise dissimilarities. This approach is ubiquitous in microbiome studies, where ecologists have provided dissimilarity measures such as Bray-Curtis, UniFrac, or Aitchison dissimilarities that allow ecologically meaningful comparisons between samples. Unfortunately, reducing data in this way can make standard analyses more difficult. For example, regression analysis typically posits the effect of covariates at the individual level, not on pairs of individuals. Interestingly, there are circumstances where it is still possible to conduct standard analyses on the paired data. For instance, if the matrix of pairwise dissimilarities is Euclidean (i.e., has only non-negative eigenvalues), then PERMANOVA (Anderson 2001) can be used to calculate F-tests for the effect of covariates in a regression setting. However, if the dissimilarity matrix is non-Euclidean (e.g., Bray-Curtis dissimilarity applied to count data), then these F-statistics can be negative. Another challenge with reducing data to pairwise dissimilarities is that PERMANOVA (Anderson 2001) is sensitive to both location shifts and changes in dispersion (scale) (Warton et al. 2012). Thus, using PERMANOVA alone, it is often difficult to determine whether observed differences between groups—or along a covariate gradient—are due to location, dispersion, or some combination of both. Therefore, it is of interest to develop tests of pure dispersion for pairwise dissimilarity data, to better understand how groups may actually differ. Traditionally, tests for pure dispersion have been built using within-group distances/dissimilarities (O’Brien 1992, Anderson 2006, Gijbels and Omelka 2013) and this is the approach we follow in this work.

In the simplest case, where we compare two or more groups, and where the dissimilarity is Euclidean, standard multidimensional scaling techniques can be used to represent the data as points in a Euclidean space centered about a common zero. Then, the Euclidean distance between each point and the mean (or median) of its group can be evaluated directly using Levene’s test for equal variances (Brown and Forsythe 1974). This is the approach taken by PERMDISP (Anderson 2006) and implemented in the betadisper function in the VEGAN R package. For non-Euclidean dissimilarities, Anderson proposes a somewhat heuristic modification to the distance to the mean (or median), but if this approach is used, it is not completely clear what is being tested.

Gijbels and Omelka (2013) developed a test for dispersion for pairwise dissimilarity data that allows valid comparisons of within-group variances when the dissimilarity is non-Euclidean. Most notably, this approach does not require the data be representable in a Euclidean space, but instead computes the mean of all pairs of within-group distances. For a Euclidean matrix, it is known that the sum of squared distances is equivalent to the mean of squared sums of distances from each observation to its group centroid, and also corresponds to the *U*-statistic form of the standard variance. When the matrix is non-Euclidean, as with Bray–Curtis dissimilarities or other abundance-type measures, the usual equivalence with sums of squared deviations from centroids no longer holds, and the formulation using pairwise distances is advantageous in both computation and interpretation. They also give a version for use with the spatial median, but most importantly, they give a clever permutation scheme that allows generation of null replicates with equal dispersion that accounts for differences in location. Gijbels and Omelka propose two tests that we denote GO (calculates p-values using an asymptotic distribution) and GO.perm (calculates p-values by permutation).

Both Anderson and Gijbels and Omelka only consider measures of dispersion characterized by a single parameter. However, differences in dispersion may be more complex than can be characterized by a single parameter; for example, two groups can have very similar *mean* dispersion even while the *distribution* of pair-wise distances can be different. Such differences in dispersion would not be detected using a test that only compares mean square distances. Here, we develop the Distance-based Test of Homogeneity (DTH) that is sensitive to arbitrary differences in dispersion (as measured by the distribution of within-group distances) between multiple groups, or across values of a continuous variable. DTH may be viewed as a direct extension of the test proposed in Gijbels and Omelka, but geared towards a broader class of alternatives.

The remainder of this work is organized as follows. In Section 2 we introduce two motivating examples; in Section 3, we describe DTH and the permutation scheme we use to assess significance. In Section 4 we describe simulations we undertook to compare DTH with the methods of Anderson and Gijbels and Omelka, concentrating on situations where differences in dispersion occur in the distribution of distances, rather than differences in a single parameter. In Section 5 we test for differences in dispersion of the oral microbiome of 511 participants by smoking status (never, former and current smokers) and by HIV status (HIV positive or negative). Finally, Section 6 contains concluding remarks.

## 2 Motivating examples

We give three examples of situations where testing for dispersion is important. Two of them are cases where tests that are sensitive to more than mean square distances are important, the other is an example of ecological data where PERMANOVA is non-significant, but tests for dispersion are.

**Figure 1:**
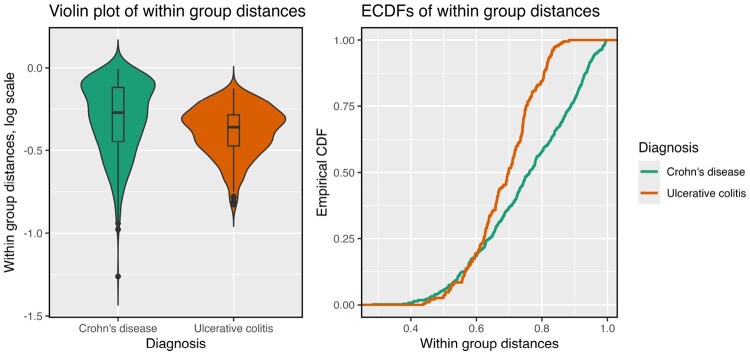
Violin plot and ECDF (empirical cumulative distribution function) of within-group distances for the two diagnosis groups (Crohn’s disease, Ulcerative Colitis) in data on 52 patients with IBD.

The first example is from gut microbiome data on patients with inflammatory bowel disease (IBD) (Lloyd-Price et al. 2019). Specifically, we consider baseline data from stool microbiome data among 32 participants diagnosed with Crohn’s Disease (CD) and 20 participants diagnosed with Ulcerative Colitis (UC). It is of interest to see how the gut microbiome differentiates these two disease categories that are typically diagnosed by clinical symptoms. In Figure 1, we show violin plots of the (log-transformed) within-group distances calculated using the Bray-Curtis distance, and also show the empirical distribution functions of the (untransformed) distances. Even on the log scale, the group medians are quite similar; however, there is clearly more dispersion in the CD group than the UC group. This is also seen in the two empirical distribution functions. In this example, we see that neither betadisper (p-value: 0.125) nor GO or GO.perm (p-values: 0.104 and 0.117 respectively) find a significant difference in dispersion, while DTH does (p-value: 0.024).

**Figure 2:**
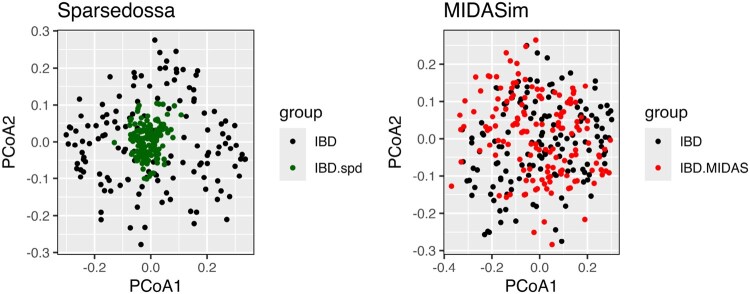
PCoA plots for Jaccard distance on the real IBD data (black) vs IBD as generated using SparseDossa (green, left panel) and MIDASim (red, right panel), respectively. Reproduced from He et al. (2024).

A second example arises when comparing methods for simulating synthetic microbiome data using the entire longitudinal IBD data as template, i.e the IBD data containing microbiome data from all visits of all patients. When developing the MIDASim (He et al. 2024) method, we noticed that both MIDASim and the SparseDossa (Ma et al. 2021) methods had mean square distances matching the template data they were designed to replicate. However, the SparseDossa data had a much narrower distribution of pairwise distances than the MIDASim data, which better matched the template data. Figure 2 offers a visual perspective of the problem, and Table 1 reports the corresponding p-values.

**Table 1:** P-values testing dispersion homogeneity between true microbiome samples from a study of IBD and simulated datasets generated by SparseDossa and MIDASim using the true samples as templates.

Method	IBD vs SparseDossa(IBD)	IBD vs MIDASim(IBD)
GO	0.987	0.613
GO.perm	0.991	0.62
betadisper	0.903	0.614
PERMANOVA	0.894	1
DTH	0.001	0.458

**Figure 3:**
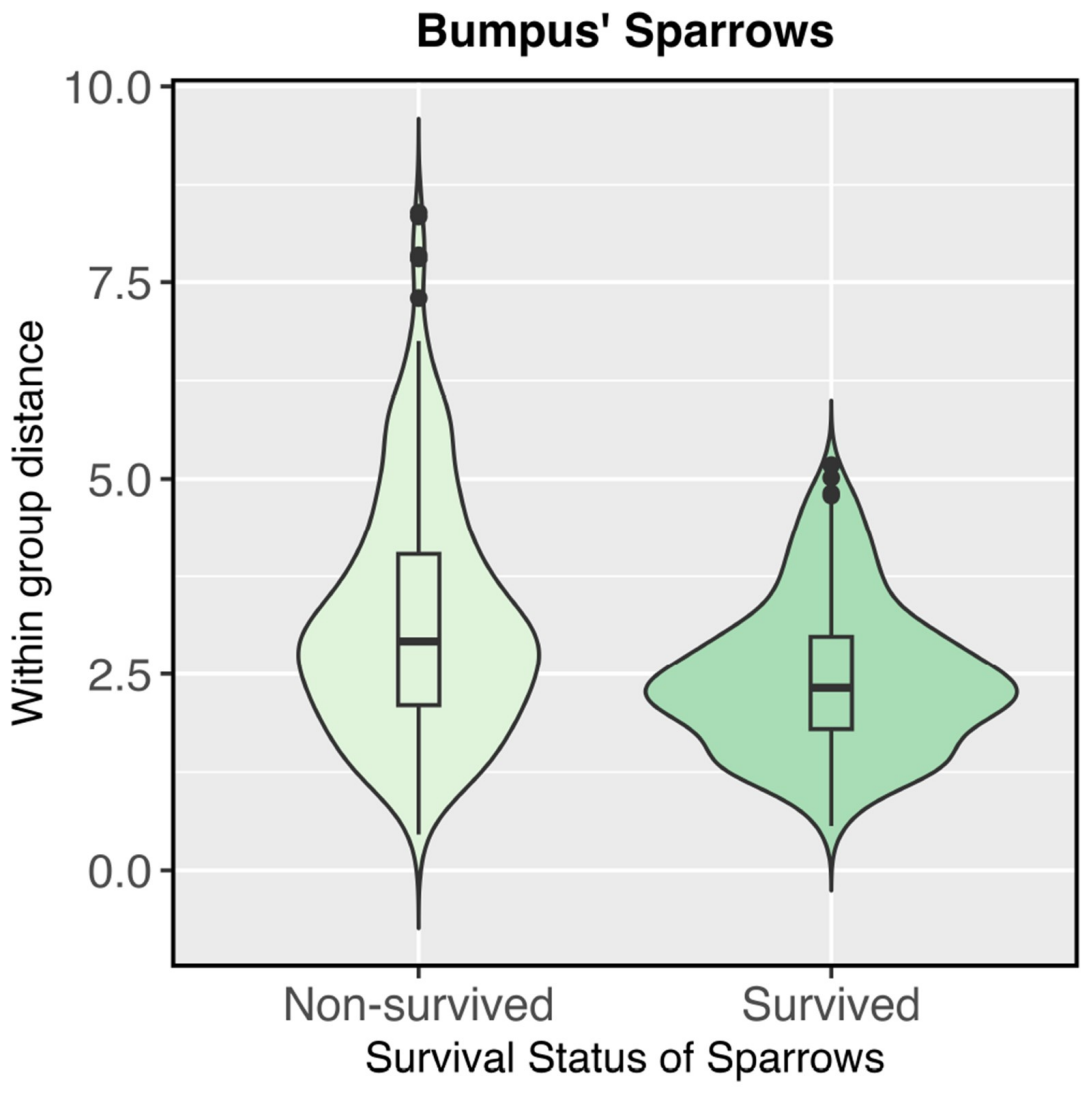
Distribution of within-group differences for the characteristics of species of sparrows by survival status. Test *p*-values: GO: 0.043, GO.perm: 0.057, PERMANOVA: 0.839, Betadisper: 0.065, DTH: 0.041.

In the third example, we consider Bumpus’ Sparrows Data (Bumpus 1899) which consists of five morphological characteristics of sparrows measured in Rhode Island, grouped by survival status after a severe storm. We consider a subset of the data considered in Manly (2005) and Gijbels and Omelka (2013), and test for differences in dispersion amongst morphological characteristics by group. It is evident from Figure 3 that there is a significant difference in the dispersion of morphological characteristics between species that survived vs species that didn’t, and this is backed up by the p-values (in the caption of the figure). The purpose of this example is to demonstrate the applicability of our proposed test beyond high dimensional data, and to give an example where between-group differences are detected using a test of dispersion, but not by PERMANOVA.

## 3 Methods

We assume that the data are summarized in a n×d matrix *Y* such that each row corresponds to an observation and each column to a feature (e.g., a taxon). We let $Y_{i\cdot }$ denote the *i*th row of *Y*. These samples are organized into *G* groups; we let $g_{i}$ denote the group that the *i*th observation belongs to. The *g*th group contains $\sum_{i}I(g_{i}=g)=n_{g}$ samples and $\sum_{g=1}^{G}n_{g}=n$. In this section, we will first consider scenario when we have samples from G=2 groups. We will discuss how to extend this approach to three or more groups in Section 3.3.

For G=2, let $L_{1}$ and $L_{2}$ denote the distributions of the within group distances for groups 1 and 2, respectively. We are interested in the following broad class of null hypothesis considered by Gijbels and Omelka (2013), namely:


(1) $$H_{0}:L_{1}=L_{2}\;\;.$$

Define the distance or dissimilarity matrix as Φ, where the ${\left(i,j\right)}^{th}$ entry is the distance between the $i^{th}$ and $j^{th}$ sample, i.e


(2) $$\phi_{ij}=\phi (Y_{i\cdot },Y_{j\cdot })\;\;,$$

where ϕ denotes the dissimilarity measure. This may be a Euclidean distance, or for microbiome data, it can be the Bray-Curtis or Jaccard dissimilarity. Following Dubey et al. (2024) we can define for the $i^{th}$ sample in the $g^{th}$ group (g=1,2)


$$F_{i}^{g}(z)=\frac{1}{n_{g}-1}\sum_{\left\{j:j\neq i\right\}}I(\phi_{ij}\leq z,g_{j}=g,g_{i}=g)\;\;,$$

which is the proportion of samples in group *g* which is within distance *z* of the $i^{th}$ observation. The mean of this quantity, taken over samples in group *g*, is


(3) $${\bar{F}}^{g}(z)=\frac{1}{n_{g}}\sum_{\left\{i:g_{i}=g\right\}}F_{i}^{g}(z)$$

(4) $$=\frac{2}{n_{g}(n_{g}-1)}\sum_{i}\sum_{j>i}I(\phi_{ij}\leq z,g_{i}=g,g_{j}=g)\;\;.$$

Considering this quantity as the building block of our test statistic instead of mean within group distance allows us to quantify the overall difference in distribution instead of the differences in mean dispersion only, hence paving the way for a broader class of hypothesis. Note that $0\leq {\bar{F}}^{g}(z)\leq 1$ is an increasing step function in *z* with up to $\left(\begin{array}{c} n_{g}\\ 2 \end{array}\right)$ jumps. Testing for equality of $L_{1}$ and $L_{2}$ translates to testing the equality of the functions ${\bar{F}}^{1}(z)$ and ${\bar{F}}^{2}(z)$. The extent of similarity between ${\bar{F}}^{1}(z)$ and ${\bar{F}}^{2}(z)$ can be measured by either the Kolmogorov-Smirnov distance (Kolmogorov 1933) or the 1-Wasserstein Distance (Vallender 1974; Kantorovich 1960) between $\Phi_{1}$ and $\Phi_{2}$, where


(5) $$\Phi_{g}:=\{\phi_{ij}:g_{i}=g,g_{j}=g,i<j\}.$$

It is known that the Kolmogorov-Smirnov test suffers from low power against all but location scale alternatives, (Fan 1996, Zhou et al. 2017), while Wasserstein distance is more powerful against smaller geometric differences (e.g outliers, differences in tail behavior) between the two distributions (Raghvendra et al. 2024). In our motivating examples and applications, we encountered both types of differences between the groups; thus, we propose an omnibus test combining the Kolmogorov-Smirnov and Wasserstein distances. This results in a test that is powerful against both location-scale and tail-behavior alternatives.

### 3.1 DTH for two groups

For each group we compute ${\bar{F}}^{g}(r)$, g=1,2, and measure the Kolmogorov-Smirnov (K) and 1-Wasserstein (W) distance between ${\bar{F}}^{1}(r)$ and ${\bar{F}}^{2}(r)$. Since ${\bar{F}}^{g}$ is a step function, both *W* and *K* can be easily calculated. We used the function wasserstein1d in the R package transport (Schuhmacher et al. 2020) to calculate *W* and the R function ks.test to calculate *K*. The usual asymptotic null distributions that are based on independent observations cannot be used to assess the significance of *W* and *K*, as the within group distances are not generally independent. Thus, to estimate the null distributions of *W*, *K* and their combinations we used the permutation approach described in Gijbels and Omelka (2013) to generate *R* replicate distance matrices $\Phi^{\left(r\right)}$ having elements $\phi_{ij}^{\left(r\right)}$ for which the dispersion is the same in each group. The corresponding set of within group distances (as in (5)) for group *g* is denoted by $\Phi_{g}^{\left(r\right)}$.

We let $K_{0}$ ($W_{0}$) denote the value of *K* (*W*) for the original data, where $K_{0}:=K(\Phi_{1},\Phi_{2})$ and $W_{0}:=W(\Phi_{1},\Phi_{2})$,

And denote by $K_{r}$ ($W_{r}$) the value of *K* (*W*) statistic for the $r^{th}$ permutation replicate for r=1…R, i.e $K_{r}:=K(\Phi_{1}^{\left(r\right)},\Phi_{2}^{\left(r\right)})$ and $W_{r}:=W(\Phi_{1}^{\left(r\right)},\Phi_{2}^{\left(r\right)})$.

The *p*-value $p_{0}^{K}$ ($p_{0}^{W}$) for the *K* (*W*) statistic is given by $p_{0}^{K}=\frac{1+\sum_{r=1}^{R}I(K_{r}>K_{0})}{R+1}$; $p_{0}^{W}$ is obtained by replacing *K* with *W*. Note that *p*-values for the null replicates can also be assigned using $p_{r}^{K}=\text{rank}(-K_{r})/R$ and $p_{r}^{W}=\text{rank}(-W_{r})/R$. The denominator is *R* as we are comparing each null replicate to the remaining R−1 null replicates.

We can combine the p-values from the K and W tests to form an omnibus test. Based on simulation results (not shown) we used


$$m=1-\min(p^{K},p^{W})$$

To combine the K and W tests, resulting in a statistic where larger values imply evidence against null. Using the *p*-values $p_{r}^{K}$ and $p_{r}^{W}$ we can calculate the value of $m_{r}=1-\min(p_{r}^{K},p_{r}^{W})$ for the original data (r=0) and each null replicate (1≤r≤R). When there are only two groups, we can then obtain $p_{0}^{m}$, the *p*-value of the omnibus test, using $p_{0}^{m}=\frac{1+\sum_{r=1}^{R}I(m_{r}>m_{0})}{R+1}$.

### 3.2 DTH for more than two groups

If G>2, i.e. if we have more than two groups, we compute the omnibus statistic $m_{0;gg^{\prime }}$ for the original data and null replicates $m_{r;gg^{\prime }}$ for each pair of groups $g,g^{\prime }$ as described in Section 3.1. We then assign *p*-values $p_{r}^{m}(g,g^{\prime })$ to each null replicate $m_{r;gg^{\prime }}$ using their rank among the null replicates as described previously. Based on simulations (results not shown), we combine the $\left(\begin{array}{c} G\\ 2 \end{array}\right)$  *p*-values $p_{r}^{m}(g,g^{\prime })$ values using Fisher’s combination statistic $f_{0}=-\sum_{g=1}^{G-1}\sum_{g^{\prime }=g+1}^{G}ln(p_{0}^{m}(g,g^{\prime }))$. Values of $f_{r}$ for each null replicate can be obtained by replacing $p_{0}^{m}(g,g^{\prime })$ by $p_{r}^{m}(g,g^{\prime })$ in the expression for $f_{0}$. The final significance is based on $p_{0}^{F}=\frac{1+\sum_{r=1}^{R}I(f_{r}>f_{0})}{R+1}$. We note that we could have combined the $p_{0}^{m}(g,g^{\prime })$  *p*-values using the Cauchy Combination test (Liu and Xie (2020)) which allows an analytic *p*-value to be assigned, but found the Fisher combination had better performance and only adds a trivial amount of computational effort. Our permutation scheme is described in Algorithm 1, which uses the more general *G*-group notation of this section even when G=2.

### 3.3 Extension to continuous variables

Although the methods developed up to this point were designed for grouped data, they can be directly applied to test the association of changes in dispersion with continuous covariates. Since our interest is in hypothesis testing, not estimation, we can discretize any continuous covariates and then test for association between the covariate-defined groups and dispersion. In fact, this approach can also be used for the GO and GO.perm approaches (Gijbels and Omelka 2013).

## 4 Simulations

We conducted simulations to assess the performance of DTH in evaluating differences in dispersion and to compare it with existing tests of differential dispersion. First, to ensure precise control over dispersion parameters, we generated multivariate data from multiple parametric distributions for *G* groups.

Algorithm 1 Workflow of DTH **Input:** Data matrix $Y\in R^{n\times d}$, group identifiers $g_{1},\ldots ,g_{n}$, number of permutations *R*, and distance ϕ(·,·).

**Output:** DTH p-value.

 **Step 1: Compute within-group distances for all groups** 

 **for**  g=1 to *G* **do**

$\Phi_{g}\leftarrow \{\phi (Y_{i\cdot },Y_{j\cdot }):g_{i}=g,\;g_{j}=g,\;i<j\}$

 **end for** 

 **Step 2: For each distinct pair of groups, compute KS and Wasserstein distances (Original Data)** 

 **for all**  $1\leq g<g^{\prime }\leq G$  **do**

$K_{0}(g,g^{\prime })\leftarrow K(\Phi_{g},\Phi_{g^{\prime }})$

$W_{0}(g,g^{\prime })\leftarrow W(\Phi_{g},\Phi_{g^{\prime }})$

 **end for** 

 **Step 3: For each of *R* permutations, compute replicate statistics** 

 **for**  r=1 to *R* **do** 

 Generate permuted replicate distance sets ${\left\{\Phi_{g}^{\left(r\right)}\right\}}_{g=1}^{G}$

  **for all**  $1\leq g<g^{\prime }\leq G$  **do**

$K_{r}(g,g^{\prime })\leftarrow K(\Phi_{g}^{\left(r\right)},\Phi_{g^{\prime }}^{\left(r\right)})$

$W_{r}(g,g^{\prime })\leftarrow W(\Phi_{g}^{\left(r\right)},\Phi_{g^{\prime }}^{\left(r\right)})$

  **end for** 

 **end for** 

 **Step 4: For each pair of groups, compute permutation *p*-values for KS and Wasserstein** 

 **for all**  $1\leq g<g^{\prime }\leq G$  **do**

$\begin{array}{c} p_{0}^{K}(g,g^{\prime })\leftarrow \frac{1+\sum_{r=1}^{R}I\{K_{r}(g,g^{\prime })>K_{0}(g,g^{\prime })\}}{R+1}\\ p_{0}^{W}(g,g^{\prime })\leftarrow \frac{1+\sum_{r=1}^{R}I\{W_{r}(g,g^{\prime })>W_{0}(g,g^{\prime })\}}{R+1} \end{array}$

  **for**  r=1 to *R* **do**

$\begin{array}{c} p_{r}^{K}(g,g^{\prime })\leftarrow \text{rank}(-K_{r}(g,g^{\prime }))/R\\ p_{r}^{W}(g,g^{\prime })\leftarrow \text{rank}(-W_{r}(g,g^{\prime }))/R \end{array}$

  **end for** 

 **end for** 

 **Step 5: For each pair of groups (Original Data and Permutations), compute omnibus statistic** 

 **for**  r=0 to *R* **do** 

  **for all**  $1\leq g<g^{\prime }\leq G$  **do**

$m_{r;gg^{\prime }}\leftarrow 1-\min\{p_{r}^{K}(g,g^{\prime }),p_{r}^{W}(g,g^{\prime })\}$

  **end for** 

 **end for** 

 **if**  G=2  **then** 

  **return**  $p\leftarrow \frac{1+\sum_{r=1}^{R}I\{m_{r;12}>m_{0;12}\}}{R+1}$

 **else** 

  **Step 6: For each pair of groups, compute permutation *p*-values for omnibus statistic** 

  **for all**  $1\leq g<g^{\prime }\leq G$  **do**

$p_{0}^{m}(g,g^{\prime })\leftarrow \frac{1+\sum_{r=1}^{R}I\{m_{r;gg^{\prime }}>m_{0;gg^{\prime }}\}}{R+1}$

  **for**  r=1 to *R* **do**

$p_{r}^{m}(g,g^{\prime })\leftarrow \text{rank}(-m_{r;gg^{\prime }})/R$

   **end for** 

  **end for** 

  **Step 7: Combine pairwise omnibus *p*-values using Fisher’s combination statistic** 

  **for**  r=0 to *R* **do**

$f_{r}\leftarrow -\sum_{g=1}^{G-1}\sum_{g^{\prime }=g+1}^{G}\ln(p_{r}^{m}(g,g^{\prime }))$

  **end for** 

  **return**  $p\leftarrow \frac{1+\sum_{r=1}^{R}I\{f_{r}>f_{0}\}}{R+1}$

 **end if** 

In the interest of fairness, we consider up to four scenarios in each setting, ranging from scenarios that favor DTH to scenarios that favor existing methods. Additionally, we performed simulations using MIDASim and sparseDOSSA, two recently-developed programs designed to generate realistic microbiome data. Both programs were designed to replicate key characteristics of real datasets, including high sparsity and large overdispersion. As shown in our motivating examples, these two methods can generate datasets with similar centroids but different dispersions. We also implement a simulation where the dispersion of the outcome is related to a continuous covariate, and report the results in the Supplementary materials (Figure A3, available as supplementary data at *Bioinformatics* online).

### 4.1 Simulation 1: multivariate normal distribution

We first simulated data from *d*-dimensional mixtures of normal distributions for G(G=2,3,5) groups with identical group means but distinct distributions of dispersions. For each sample *i* in group *g*, we generate $Y_{i\cdot }|v_{i}∼N_{d}(0_{d},v_{i}I_{d})$, where $v_{i}$ is generated from a mixture distribution, and let the parameters of the mixture vary from one group to another. We chose d=500 for all simulations, and measured distance using the Euclidean distance. We simulated four scenarios corresponding to four choices for the mixture distribution of $v_{i}$ denoted by S0, S1, S2, and S3. These scenarios are ordered to grade progressively from scenarios that favor DTH (S0, S1) to situations that favor existing tests (S2, S3). In S0 we choose $v_{i}∼\text{Gamma}(\alpha_{g_{i}},\kappa_{g_{i}})$ so that $E(v_{i})=\alpha_{g_{i}}\kappa_{g_{i}}$. We choose $\alpha_{g}$ and $\kappa_{g}$ so that $\frac{\Gamma (2\alpha_{g}+\frac{1}{2})}{\Gamma (2\alpha_{g})}\sqrt{\kappa_{g}}=1\;\;\forall g$; with this choice, $E(\phi_{ii^{\prime }})$=Const when $g_{i}=g_{i^{\prime }}$ so that the GO tests and betadisper should have negligible power. In S1 and S2 we choose $v_{i}∼\Lambda (\mu_{g_{i}},\sigma_{g_{i}}^{2})$ where Λ denotes the log normal distribution, so that $E(v_{i})=\text{exp}(\mu_{g_{i}}+\sigma_{g_{i}}^{2}/2)$. In S1 we choose $\mu_{g}+\frac{1}{2}\sigma_{g}^{2}=\frac{3}{2}\;\;\forall g$ so that the mean dispersion $E(v_{i})$ is constant across groups. Because the distribution of the dispersion differs between groups, this scenario favors our method, which is sensitive to differences in the distribution of dispersions. In S2 we keep $\mu_{g}$ constant ∀g, and vary $\sigma_{g}^{2}$ by group, so that the mean dispersion $E(v_{i})$ also varies across groups. S2 favors existing methods that are designed to detect differences in mean dispersion. In S3, we choose a fixed value $v_{g}$ for each group (see Table 2 for the values). We consider both balanced (B) scenarios, where each group has the same number of samples, and unbalanced scenarios (UB), where group sizes differ. Results for balanced settings are presented in the main paper; results for unbalanced settings are given in the Supplementary materials (Figure A1, available as supplementary data at *Bioinformatics* online). The values of the mixing distribution parameters and sample sizes for each simulation are given in Table 2. Each parameter is expressed as a function of θ, a parameter which controls the deviation from the null hypothesis. In all cases, θ=0 corresponds to the null hypothesis that the distribution of *Y* is identical in each group.

**Table 2:** Simulation parameters for Simulations 1 & 2.

G	Multivariate Normal Parameters	Negative Binomial Parameters
2	**S0**: $(\alpha_{1},\alpha_{2})=(1,1+3\theta );\;\kappa_{i}={\left(\frac{\Gamma (2\alpha_{i}+\frac{1}{2})}{\Gamma (2\alpha_{i})}\right)}^{2}\forall i$ **S1**: $(\mu_{1},\mu_{2})=(1,1-\theta );\;\sigma_{i}^{2}=3-2\mu_{i}\forall i$ **S2**: $\mu_{i}=1\forall i;\;(\sigma_{1}^{2},\sigma_{2}^{2})=(1,1+2\theta )$ **S3**: $\left(v_{1},v_{2})=(1,\;\text{exp}\;\frac{\theta }{80}\right)$	**S1**: $(\alpha_{1},\alpha_{2})=5\cdot (1,\;\text{exp}(-\theta ));\beta_{i}=\alpha_{i}$ **S2**: $\left(\alpha_{1},\alpha_{2})=(3,3);(\beta_{1},\beta_{2})=5\cdot (1,\;\text{exp}(-\frac{\theta }{10})\right)$ **S3**: $\left(r_{1},r_{2})=(1,\;\text{exp}(\frac{\theta }{30})\right)$
3	**S0**:$(\alpha_{1},\alpha_{2},\alpha_{3})=(1,1+6\theta ,1+3\theta );\;\kappa_{i}=\left(\frac{\Gamma (2\alpha_{i}+\frac{1}{2})}{\Gamma (2\alpha_{i})}\right)\forall i$ **S1**: $(\mu_{1},\mu_{2},\mu_{3})=(1,1-\frac{\theta }{2},1-\theta );\;\sigma_{i}^{2}=3-2\mu_{i}\forall i;$ **S2**: $\mu_{i}=1\forall i;\;(\sigma_{1}^{2},\sigma_{2}^{2},\sigma_{3}^{3})=(1,1+\theta ,1+2\theta )$ **S3**: $\left(v_{1},v_{2},v_{3})=(1,\;\text{exp}\;\frac{\theta }{40},\;\text{exp}\;\frac{\theta }{80}\right)$	**S1**: $(\alpha_{1},\alpha_{2},\alpha_{3})=5\cdot (1,\;\text{exp}(-\frac{\theta }{2}),\;\text{exp}(-\theta ));\beta_{i}=\alpha_{i}$ **S2**: $\left(\alpha_{1},\alpha_{2},\alpha_{3})=(3,3,3);(\beta_{1},\beta_{2},\beta_{3})=5\cdot (1,\;\text{exp}(-\frac{\theta }{10},\;\text{exp}(-\frac{\theta }{10})\right)$ **S3**: $\left(r_{1},r_{2},r_{3})=(1,\;\text{exp}(\frac{\theta }{60}),\;\text{exp}(\frac{\theta }{30})\right)$
5	**S0**: $(\alpha_{1},\ldots \alpha_{5})=(1,1+6\theta ,1,1,1+3\theta ,1);$ $\kappa_{i}={\left(\frac{\Gamma (2\alpha_{i}+\frac{1}{2})}{\Gamma (2\alpha_{i})}\right)}^{2}\forall i$ **S1**: $(\mu_{1},\ldots \mu_{5})=(1,1,1-\frac{\theta }{2},1,1-\theta );$ $\sigma_{i}^{2}=3-2\mu_{i}\forall i;$ **S2**: $\mu_{i}=1\forall i;(\sigma_{1}^{2},\ldots ,\sigma_{5}^{2})=(1,1,1+\theta ,1,1+2\theta )$ **S3**: $\left(v_{1},v_{2},v_{3},v_{4},v_{5})=(1,1,\;\text{exp}\;\frac{\theta }{40},1,\;\text{exp}\;\frac{\theta }{80}\right)$	**S1**: $(\alpha_{1},\ldots ,\alpha_{5})=5\cdot (1,1,\;\text{exp}(-\frac{\theta }{2}),1,\;\text{exp}(-\theta ));\beta_{i}=\alpha_{i}$ **S2**: $(\alpha_{1},\ldots ,\alpha_{5})=(3,3,3,3,3);$ $\left(\beta_{1},\ldots ,\beta_{5})=5\cdot (1,1,\;\text{exp}(-\frac{\theta }{20}),1,\;\text{exp}(-\frac{\theta }{10})\right)$ **S3**: $\left(r_{1},\ldots ,r_{5})=(1,1,\;\text{exp}(\frac{\theta }{60}),1,\;\text{exp}(\frac{\theta }{30})\right)$

For Multivariate Normal outcomes θ, the degree of departure from the null hypothesis, varies from 0 to 2 in increments of 0.5 in each scenario. For Negative Binomial outcomes θ, the degree of departure from the null hypothesis, varies from 0 to 3 in increments of 0.5. d=500 and $\sum_{i=1}^{G}n_{i}=150$ across all simulations.

**Figure 4:**
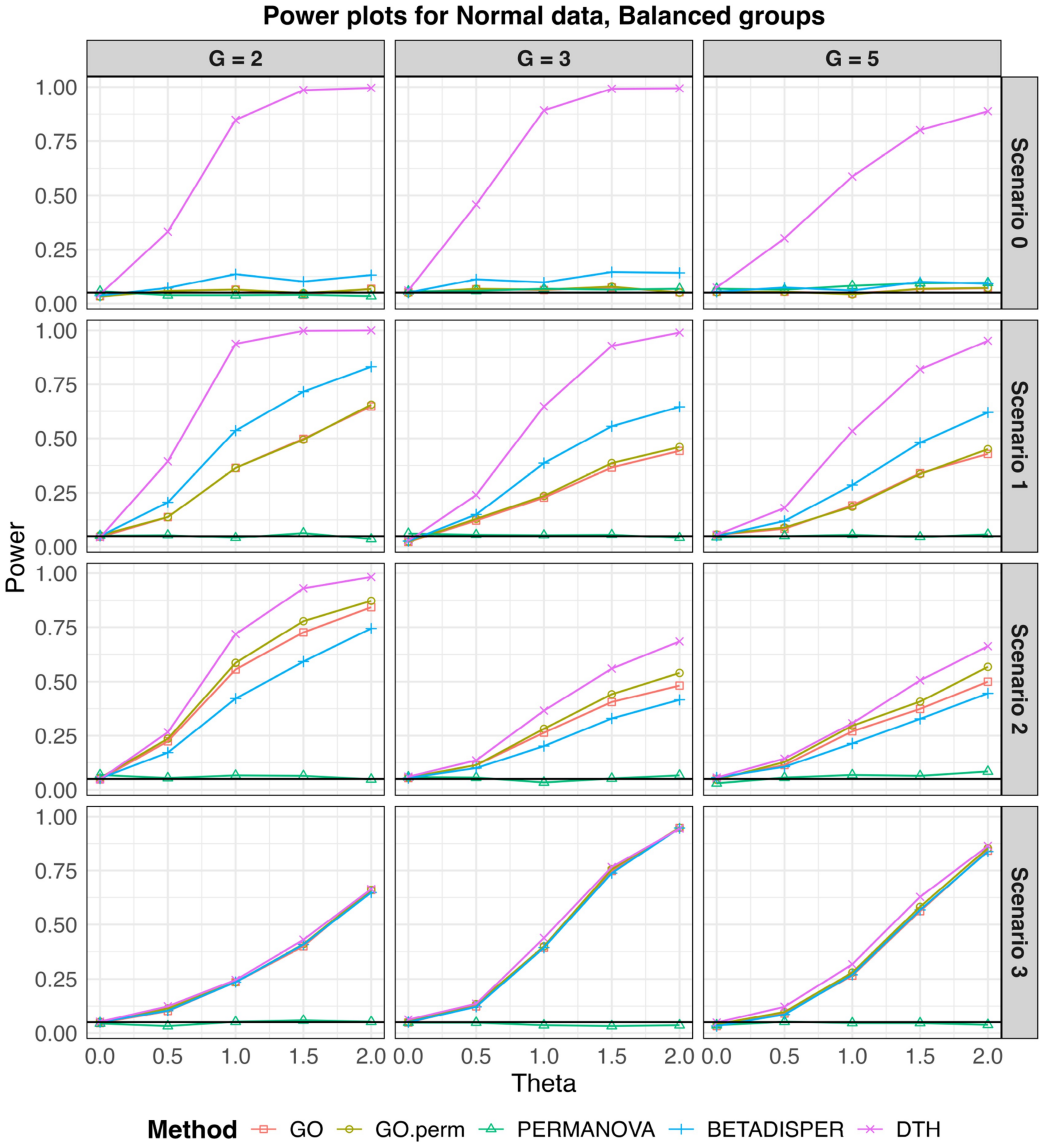
Empirical type I error and power for multivariate normal outcomes for balanced designs. Sample size for all simulations was 150 observations split evenly among the *G* (G=2,3,5) groups. The black horizontal line marks the nominal type I error level (0.05) and θ=0 corresponds to the null hypothesis in all scenarios.

In Figure 4 we show the empirical power for each test for each scenario and three values of *G*. At θ=0 we see that each test controlled the empirical size. For all simulations with normal outcomes, GO had an average size of 0.048 (range: 0.022–0.066), GO.perm had an average size of 0.049 (range: 0.024—0.066), Betadisper had an average size of 0.048 (range: 0.026—0.062) and DTH had an average size of 0.051(range: 0.032 – 0.064). A complete table of sizes for all simulations is presented in Table 1 of the Supplementary Materials, available as supplementary data at *Bioinformatics* online. In every scenario but S3, DTH had noticeably higher power than the other tests. Even in S3, DTH had comparable or slightly higher power than the other tests. Betadisper performed second best in S0 and S1, while the GO tests were second best in S2.

### 4.2 Simulation 2: negative binomial distribution

Because microbiome data are often count data, we generated data $Y_{ij}$ from a mixture of negative binomial distributions. For observation *i*, we generated data for 500 taxa using $Y_{ij}∼NB(p_{g_{i}},r_{g_{i}})$ where $r_{i}∼\mathrm{InvGamma}(\alpha_{g_{i}},\beta_{g_{i}})$. We chose NB parameter $p_{g}=r_{g}/(\mu_{g}+r_{g})$ so that $E(Y_{ij}|p_{g_{i}},r_{g_{i}})=\mu_{g_{i}}$ and $\mathrm{Var}(Y_{ij}|p_{g_{i}},r_{g_{i}})=\mu_{g_{i}}+\mu_{g_{i}}^{2}/r_{g_{i}}$; hence the unconditional moments of *Y* are $E(Y_{ij})=\mu_{g_{i}}$ and $\mathrm{Var}(Y_{ij})=\mu_{g_{i}}+\mu_{g_{i}}^{2}\left(\alpha_{g_{i}}/\beta_{g_{i}}\right)$. Here, we consider three scenarios S1-S3 that are comparable to S1-S3 for the normal simulations in 4.1. We do not include a scenario analogous to S0 as it is difficult to construct this case. Distance was measured using the Bray-Curtis distance.

**Figure 5:**
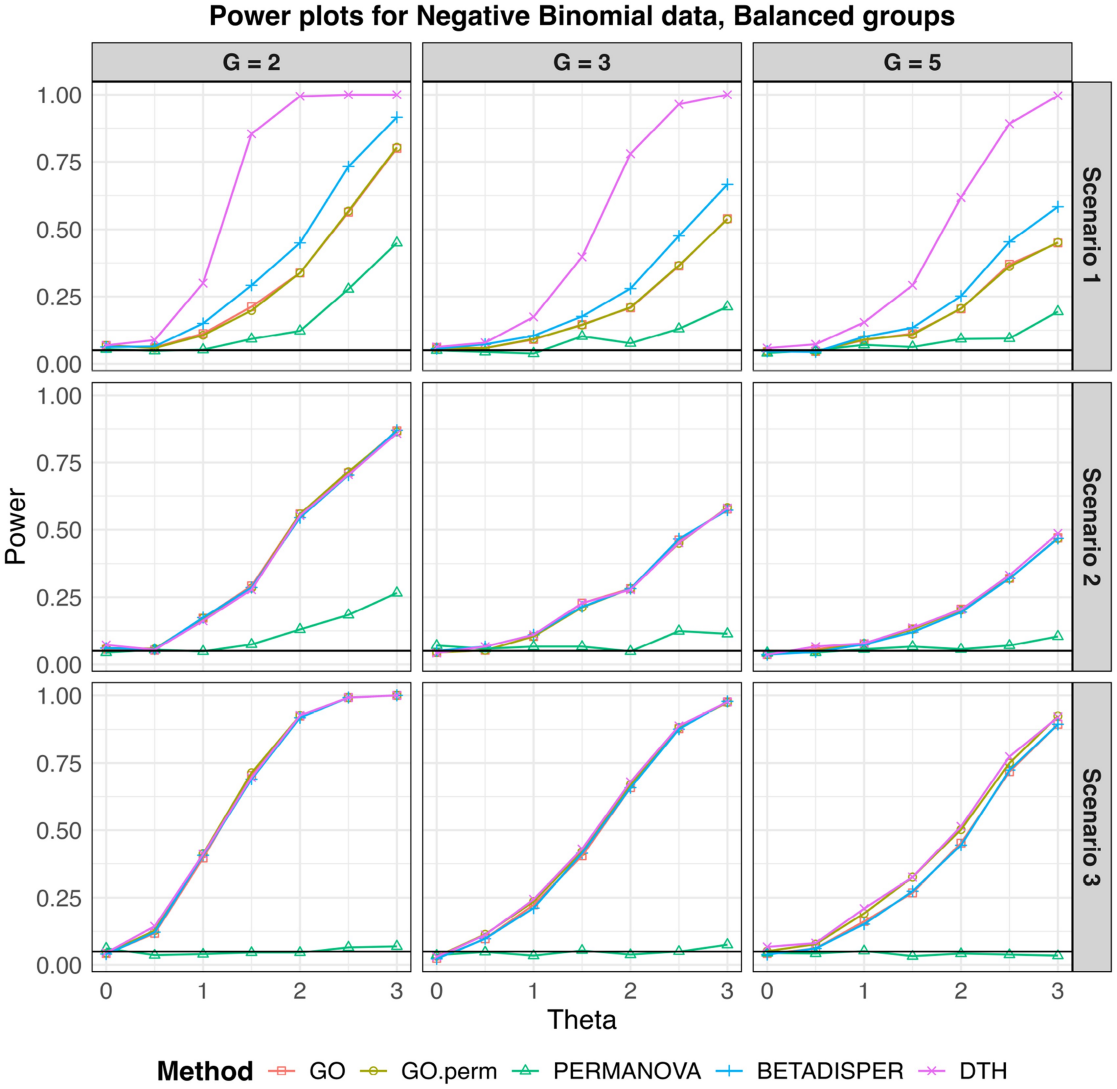
Empirical type I error and power for Negative Binomial outcome with balanced designs. Sample size for all simulations was 150 observations split evenly among the *G* (G=2,3,5) groups. The black horizontal line marks the nominal type I error level (0.05) and θ=0 corresponds to the null hypothesis in all scenarios.

For S1, we vary the parameters $\alpha_{g}$ and $\beta_{g}$ across groups such that $\frac{\alpha_{g}}{\beta_{g}}$ remains constant. This ensures that the mean dispersion remains the same in all groups but the distribution of the dispersion varies. For S2, we held $\alpha_{g}$ constant across groups while varying $\beta_{g}$. This creates a simulation setting that favors the existing methods by changing mean dispersion, i.e $E(\frac{1}{r_{g}})$ across groups. For S3, $r_{i}$, the dispersion parameter associated with the Negative Binomial Distribution is deterministic, and changes across groups in the non-null settings. The number of groups, their sizes, and parameter values as a function of a parameter θ are given in Table 2. For each scenario, we consider a balanced (B) and unbalanced (UB) setting, where the results for the balanced setting is presented in Figure 5, and the unbalanced setting is presented in the Supplementary materials (Figure A2, available as supplementary data at *Bioinformatics* online).

We find that at θ=0, all methods control empirical size. For all simulations with negative binomial outcome GO has an average empirical size of 0.048 (range: 0.024—0.074), GO.perm 0.050 (range: 0.032–0.074), Betadisper 0.0477(range: 0.022—0.072) and DTH 0.049 (range: 0.026 – 0.068). Complete table of size for all simulations is presented in Table 2 of the Supplementary Materials, available as supplementary data at *Bioinformatics* online.

When θ>0, in S1, DTH markedly outperformed the competing methods. In S2, it had comparable power to GO, GO.perm and Betadisper.

### 4.3 MOMS-PI data

**Figure 6:**
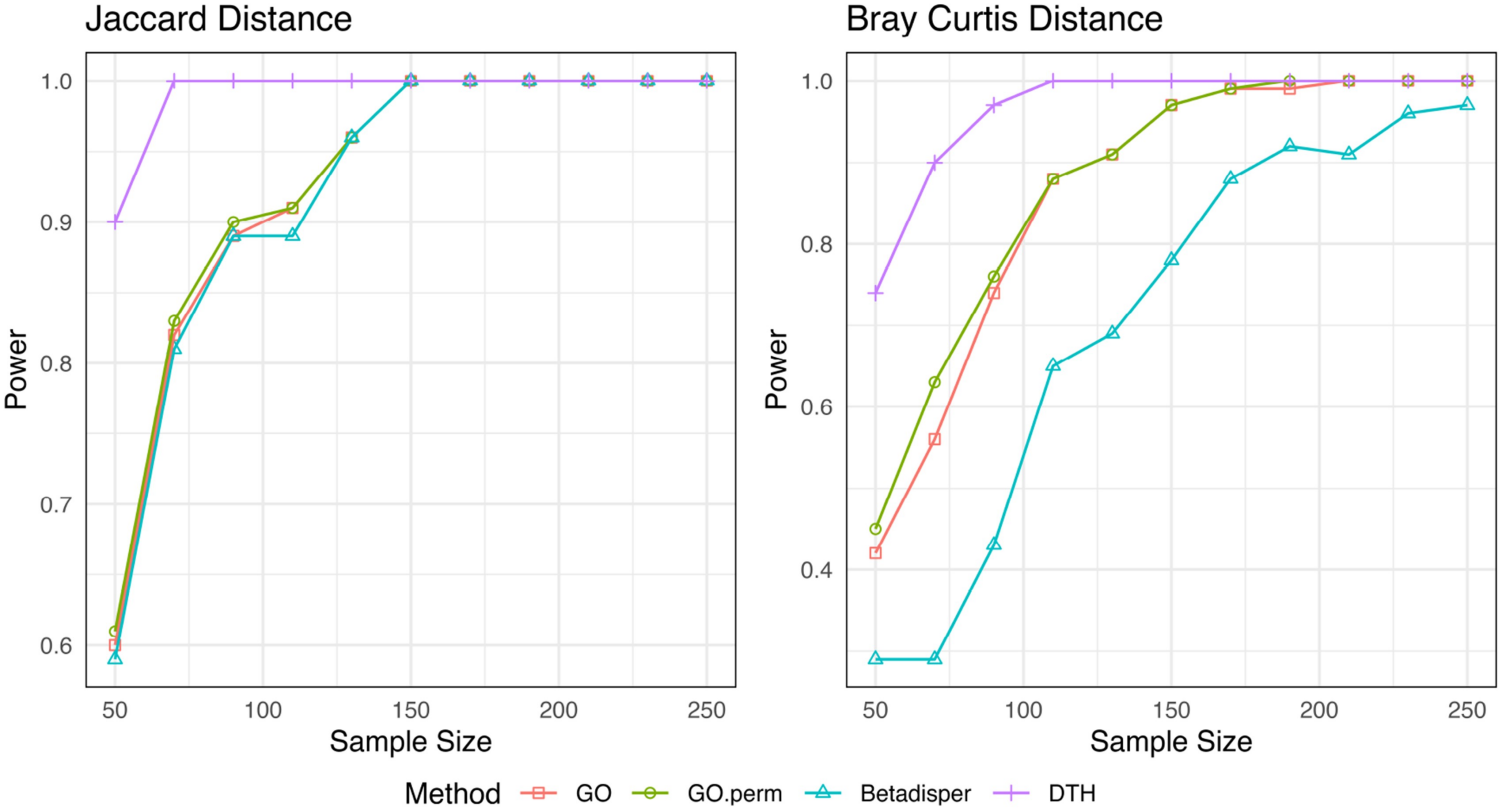
Empirical power of tests of difference in dispersion between MOMS-PI data and simulated data based on MOMS-PI but generated using sparseDOSSA. Each point is based on 1000 randomly selected subsamples from each dataset at smaller sample sizes (50 to 250). All methods have 100% power at N=150 using the Jaccard Distance, and all but Betadisper have 100% power for N>200 using the Bray-Curtis Distance. DTH has markedly higher power at smaller sample sizes.

To evaluate the performance of DTH on realistic microbiome data, we compared data from the Multi-Omic Microbiome Study–Pregnancy Initiative (MOMS-PI) (Fettweis et al. 2019) with simulated data generated using sparseDOSSA (Ma et al. 2021), which was designed to mimic the characteristics of the MOMS-PI dataset. The original MOMS-PI and simulated data each have 514 samples with 1146 taxa. Given this large sample size, all methods yield highly significant p-values (<0.001). Each observation in the original dataset has a corresponding observation in the simulated data. To generate meaningful comparisons, we randomly sampled between 50 and 250 observations from the original data as well as the corresponding observations from the simulated data, and computed p-values comparing the subsample from the original data to the corresponding subsample from the simulated data. The reported power is the proportion of subsamples that showed a significant difference (at the p = 0.05 level) out of 1000 subsamples. We used all 1146 taxa in each subsample, and all competing methods were evaluated using both the Jaccard and Bray Curtis dissimilarities. The resulting power values are reported in Figure 6. We see that DTH has higher power at smaller sample sizes, i.e. it can detect the difference between the real and simulated data at much smaller sample sizes than the other methods.

### 4.4 IBD data with MIDASim

To evaluate the performance of DTH on microbiome data under a controlled simulation setting, we simulated data based on the IBD dataset described in Section 2, which includes 32 participants with Crohn’s Disease (CD) and 20 with Ulcerative Colitis (UC). We first removed taxa that were entirely absent in either group, retaining 327 taxa in the analysis. For each simulation replicate, we used MIDASim to generate 52 CD-like microbiome profiles, using the original CD participants as the template, and 20 UC-like profiles, using the UC participants as the template. When generating simulated CD data, we preserved the original 52 observed library sizes from the 52 total participants.

To study the power of the competing methods, we generated data that smoothly transitions from CD-like to UC-like. To accomplish this, we introduced a parameter θ such that, with probability 1−θ we would replace the $i^{\text{th}}$ UC microbiome profile with the $32+i^{\text{th}}$ CD microbiome profile. This replacement was independently decided for each UC observation in each simulation replicate. Thus, when θ=0 all data are generated from the CD template, while when θ=1 all CD samples are generated from the CD template and all UC samples are generated from the UC template.

**Figure 7:**
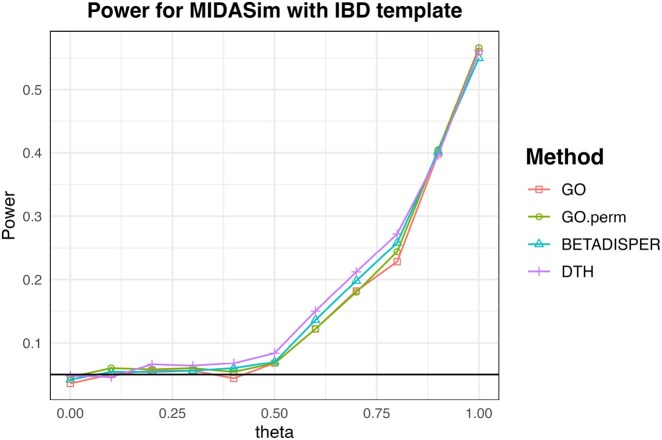
Empirical rejection rates for testing differences in dispersion between simulated CD (Crohn’s Disease) and UC (Ulcerative Colitis) microbiome profiles, simulated using MIDASim. Parameter θ=0 corresponds to no difference between CD and UC, while θ=1 corresponds to the observed difference between CD and UC in the IBD data used as a template.

The results of these simulations is shown in Figure 7. All tests have about the same power, with DTH displaying marginally improved power over betadisper, followed by the two GO tests. This indicates that even in complex microbiome data, DTH has comparable power to GO and betadisper even though it tests a much broader alternative.

## 5 Analyses of oral microbiome data

In addition to the data already analyzed in the motivating examples, we also analyzed data from the Men and Women Offering Understanding of Throat HPV (MOUTH) study (Zhang et al. 2022). Using data from this study, we compared the dispersion in oral rinse microbiome profiles between smokers and non-smokers, and persons with and without HIV infection.

**Figure 8:**
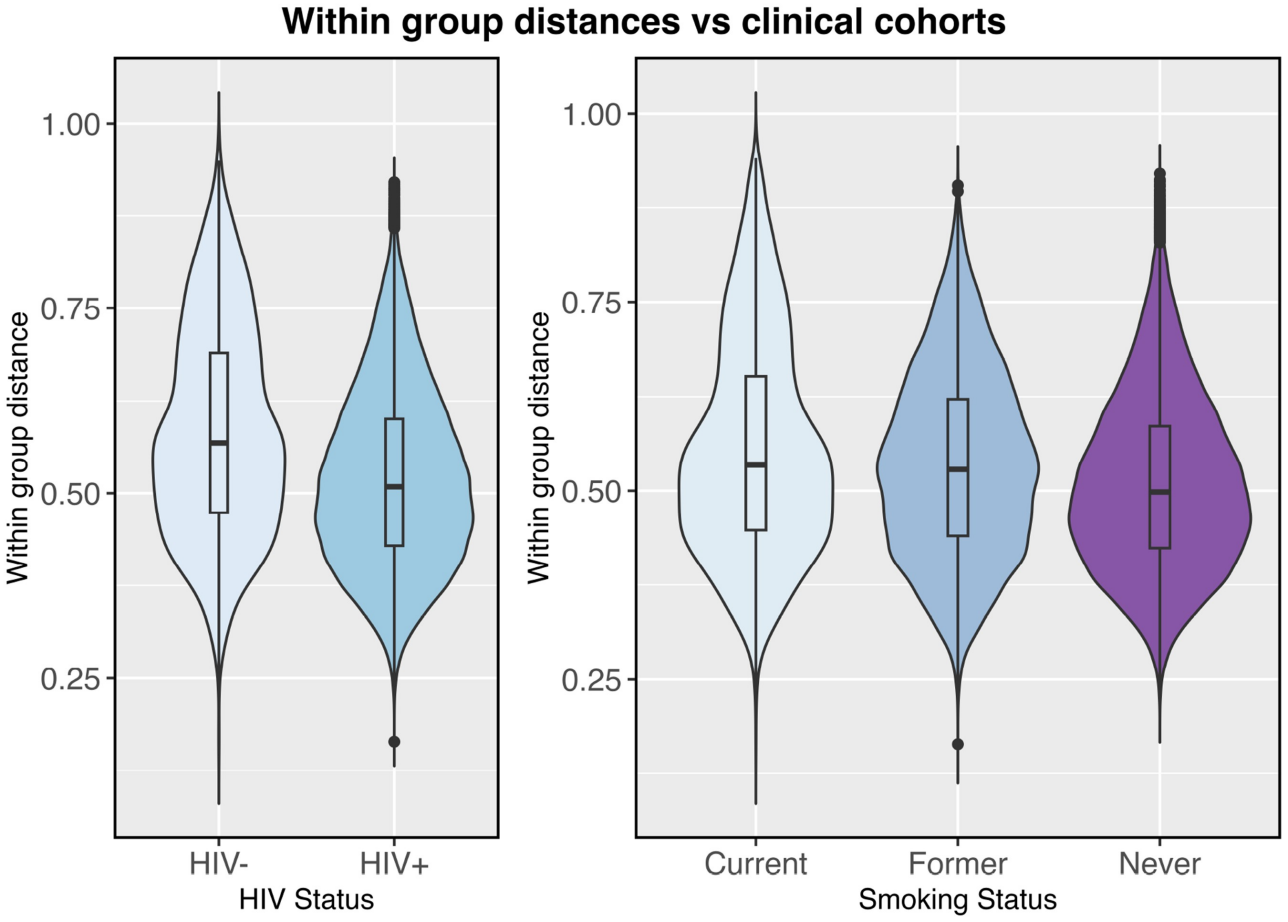
Distribution of within group distances of MOUTH study microbiome data with groups defined by clinical status (left: HIV status, right: smoking status).

For the HIV analysis, data from 524 participants were used, where 443 (84.5%) tested HIV-negative and 81 (15.5%) tested HIV-positive. For the smoking analysis, data from 511 participants were used and grouped by smoking status: never smokers (281 participants), former smokers (150 participants), and current smokers (60 participants). Violin plots depicting the distributions of the within-group distances are provided in Figure 8. The tests for differences in dispersion across smoking status yielded significant *p*-values for all methods, with DTH having the largest p-value at 0.00011 (for all other methods, p = 0.0001 except GO which only returned p<0.001). The tests for differences in dispersion across HIV status had significant p-values for all methods as well (GO: 0.006, GO.perm: 0.00071, PERMANOVA: 0.00012, betadisper: 0.00197, DTH: 0.00662). Thus, these analyses serve as additional examples where DTH performs well in real microbiome data.

## 6 Discussion

We have proposed DTH, a non-parametric test for dispersion that allows for more general alternative hypotheses than either of the two existing tests, Betadisper or the GO tests of Gijbels and Omelka (2013). Our simulations and analyses of microbiome data indicate clearly that dispersion is more complex than can be captured by a single quantity. Dispersion can act at different scales; as our simulations show, simple tests may have very different performance when $E(\phi_{ii^{\prime }})$ is similar across groups than when $E(\phi_{ii^{\prime }}^{2})$ is similar. For this reason, a test that is sensitive to broad alternatives such as DTH is favored. Furthermore, our simulations show that, at least in the cases we considered, the loss of power incurred by using such a flexible test is, at worst, small.

We have illustrated the use of DTH in a number of applications, including assessment of the performance of large-scale omics data simulators. Dispersion tests can have inherent value. For example, we may find that case probands may exhibit higher diversity than controls (which may indicate unrecognized disease subtypes) or lower diversity (which may indicate a specific risk profile). Further, testing for differential dispersion is an important step in validating the assumptions of ANOVA and PERMANOVA, where it can be useful to verify the homoscedasticity assumption.

Although the emphasis and examples in this paper have all come from microbiome and ecological studies, this is not the only situation where we may be interested in dispersion across groups. Many types of non-numeric data can be converted to pairwise distances, such as forensic databases of fingerprint similarities. For data of this kind, tests of dispersion could be informative; for fingerprint data, we may want to see if dispersion varies across ethnic groups. Our tests also apply to numeric but high-dimensional data that can be converted to distances; for example, functional data could be converted into pairwise distances, after which distance-based approaches such as DTH could be used for further analysis. Although some features of the functional data may be lost, the gain in simplicity may be worthwhile.

## Supplementary Material

btag178_Supplementary_Data

## Data Availability

All the data used in this paper and code are publicly available at https://github.com/asmita112358/DTH.

## References

[btag178-B1] Anderson MJ. A new method for non-parametric multivariate analysis of variance. Austral Ecol 2001;26:32–46.

[btag178-B2] Anderson MJ. Distance-based tests for homogeneity of multivariate dispersions. Biometrics 2006;62:245–53. 16542252 10.1111/j.1541-0420.2005.00440.x

[btag178-B3] Brown MB , Forsythe AB. Robust tests for the equality of variances. J Am Stat Assoc 1974;69:364–7.

[btag178-B4] Bumpus HC. The elimination of the unfit as illustrated by the introduced sparrow, *Passer domesticus.* In: *Biological Lectures Delivered at the Marine Biological Laboratory of Wood's Holl, 1898.* Boston: Ginn & Co, 1899, 209–226.

[btag178-B5] Dubey P , Chen Y, Müller H-G. Metric statistics: exploration and inference for random objects with distance profiles. Ann Statist 2024;52:757–92.

[btag178-B6] Fan J. Test of significance based on wavelet thresholding and neyman’s truncation. J Am Stat Assoc 1996;91:674–88.

[btag178-B7] Fettweis J , Serrano M, Brooks J et al The vaginal microbiome and preterm birth. Nat Med 2019;25:1012–21. 31142849 10.1038/s41591-019-0450-2 PMC6750801

[btag178-B8] Gijbels I , Omelka M. Testing for homogeneity of multivariate dispersions using dissimilarity measures. Biometrics 2013;69:137–45. 23002793 10.1111/j.1541-0420.2012.01797.x

[btag178-B9] He M , Zhao N, Satten GA. Midasim: a fast and simple simulator for realistic microbiome data. Microbiome 2024;12:135. 39039570 10.1186/s40168-024-01822-z PMC11264979

[btag178-B10] Kantorovich LV. Mathematical methods of organizing and planning production. Manage Sci 1960;6:366–422.

[btag178-B11] Kolmogorov A. Sulla determinazione empirica di una legge didistribuzione. Giorn Dell’inst Ital Degli Att 1933;4:89–91.

[btag178-B12] Liu Y , Xie J. Cauchy combination test: a powerful test with analytic p-value calculation under arbitrary dependency structures. J Am Stat Assoc 2020;115:393–402. 33012899 10.1080/01621459.2018.1554485 PMC7531765

[btag178-B13] Lloyd-Price J , Arze C, Ananthakrishnan AN et al Multi-omics of the gut microbial ecosystem in inflammatory bowel diseases. Nature 2019;569:655–62. 31142855 10.1038/s41586-019-1237-9 PMC6650278

[btag178-B14] Ma S , Ren B, Mallick H et al A statistical model for describing and simulating microbial community profiles. PLoS Comput Biol 2021;17:e1008913. 34516542 10.1371/journal.pcbi.1008913 PMC8491899

[btag178-B15] Manly BF. Multivariate Statistical Methods: A Primer. 3rd edn, London: Chapman & Hall/CRC, 2004.

[btag178-B16] O’Brien PC. Robust procedures for testing equality of covariance matrices. Biometrics 1992;48:819–27.

[btag178-B17] Raghvendra S , Shirzadian P, Zhang K. A new robust partial p-wasserstein-based metric for comparing distributions. In: *Proceedings of the 41st International Conference on Machine Learning, PMLR 2024*; 2024, vol. 235, 41867–85.

[btag178-B18] Schuhmacher D , Bähre B, Gottschlich C et al transport: Computation of optimal transport plans and wasserstein distances. R package version 0.15-4, 2024.

[btag178-B19] Vallender S. Calculation of the wasserstein distance between probability distributions on the line. Theory Probab Appl 1974;18:784–6.

[btag178-B20] Warton DI , Wright ST, Wang Y. Distance-based multivariate analyses confound location and dispersion effects. Methods Ecol Evol 2012;3:89–101.

[btag178-B21] Zhang Y , D’Souza G, Fakhry C et al Oral human papillomavirus associated with differences in oral microbiota beta diversity and microbiota abundance. J Infect Dis 2022;226:1098–108. 35038733 10.1093/infdis/jiac010 PMC9492316

[btag178-B22] Zhou W-X , Zheng C, Zhang Z. Two-sample smooth tests for the equality of distributions. Bernoulli 2017;23:951–989.

