# Supplementary material for: DTH: a nonparametric test for homogeneity of multivariate dispersions
Source: Bioinformatics. 2026 Apr 10;42(5):btag178. doi: 10.1093/bioinformatics/btag178 (PMC13188977; doi:10.1093/bioinformatics/btag178)
Supplement: btag178_Supplementary_Data [file btag178_supplementary_data.pdf]

Supplemental Material for "DTH: A nonparametric test for homogeneity of multivariate dispersions"

Asmita Roy[1], Jiuyao Lu, Glen A. Satten[2], Ni Zhao[1,*]

In the supplement, we present the additional simulation results for unbalanced groups for Normal and Negative Binomial data, as well as continuous covariates. We also give tables of the empirical size for all simulations.

A1 Additional simulation results: Power for Unbalanced Groups

Figures A1 and A2 recapitulate results in Figures 4 and 5, but for unbalanced group sizes. See caption of Figures A1 and A2 for group sizes. The results are similar, with DTH showing slightly less power in Scenario 3. All methods have well-controlled type I error rates, corresponding to $\theta = 0$.

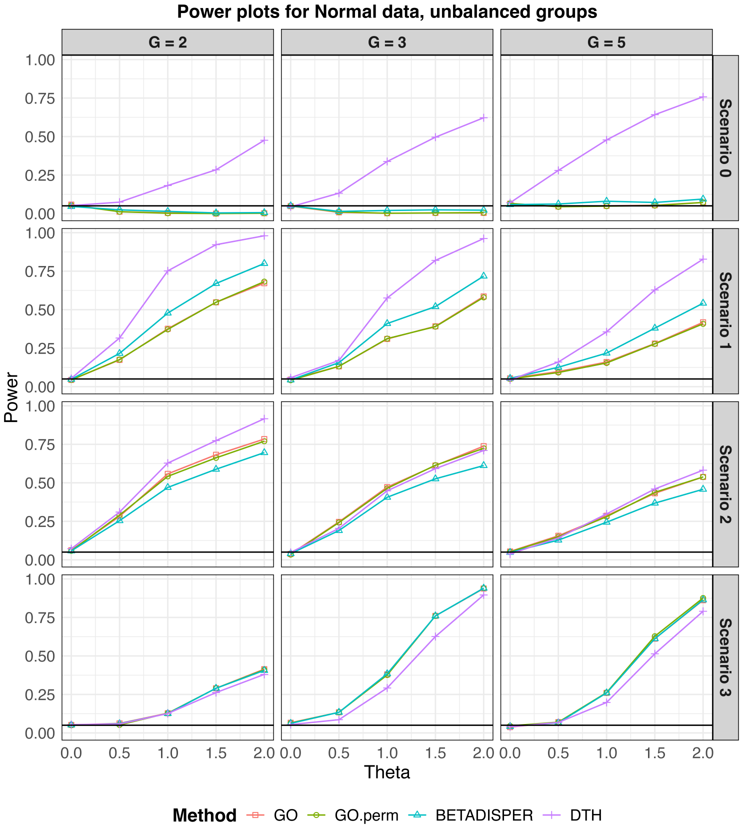

Fig. A1: Empirical type I error and power for Multivariate Normal data with unbalanced designs ($G = 2, 3, 5$). The total sample size was fixed at 150. For two-group comparisons, sample sizes were 125 and 25; for three groups, 100, 30, and 20; and for five groups, 50, 40, 30, 20, and 10. The black horizontal line marks the nominal rejection level (0.05), used to assess type I error rate control at $\theta = 0$.

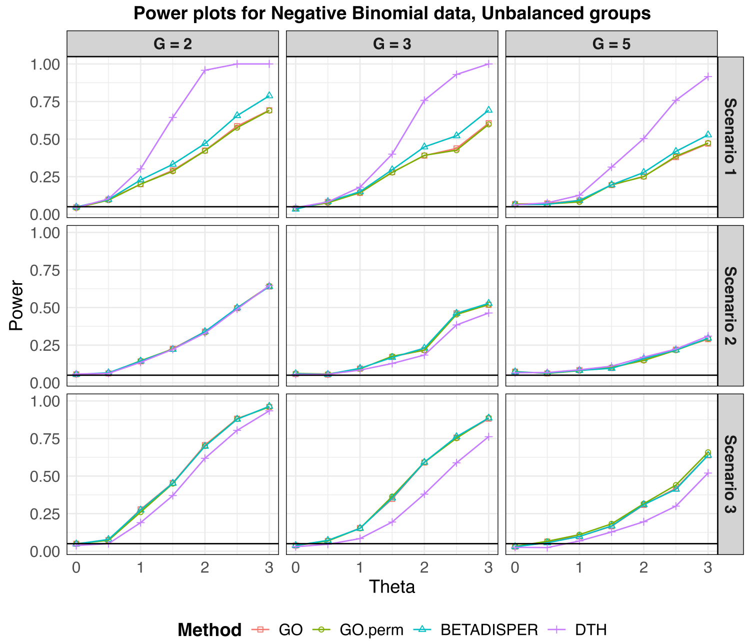

Fig. A2: Empirical type I error and power for Negative Binomial data with unbalanced designs (G = 2,3,5). The total sample size was fixed at 150. For two-group comparisons, sample sizes were 125 and 25; for three groups, 100, 30, and 20; and for five groups, 50, 40, 30, 20, and 10. The black horizontal line marks the nominal rejection level (0.05), used to assess type I error rate control at $\theta = 0$.

A2 Simulation Example with Continuous Covariates

As described in the main paper, `DTH` can also be used to test for homogeneity with respect to a continuous covariate. Here we demonstrate this using a simulation where heteroscedasticity is explained by a covariate $x_i \sim Unif(0,5)$. We generate $n = 150$ observations $Y_{i\cdot}$, each having $d = 500$ components, using the model

$$Y_{i\cdot}|v_i \sim N_d(\mathbf{0}_d, v_i\mathbf{I}_d) \quad i = 1, \cdots, n$$

where

$$v_i \sim \Lambda(-\theta x_i^2, 1 + 2\theta x_i^2) \quad i = 1, \cdots, n$$

and where $\Lambda(\mu, \sigma)$ is the log-normal distribution. This choice ensures that $\mathbb{E}(v_i) = \exp(0.5)$ independent of covariate x, while the distribution of v does depend on the covariate; thus this simulation is similar to Scenario 1 in Simulation 1 (Normal Data Simulation). The parameter θ controls the degree of heteroscedasticity; $\theta = 0$ corresponds to the null hypothesis, i.e., no change in dispersion across values of x. To test for differences in dispersion across values of x, we bin x into intervals of length 1, resulting in 5 bins, and treat each bin as a group. Power for each test considered here, for θ varying between 0 to 0.15, is reported in Figure A3. We observe that `DTH` is the most powerful amongst all competing methods.

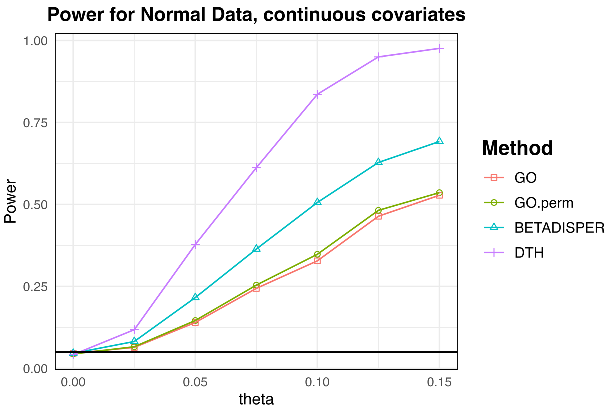

Fig. A3: Empirical type I error ($\theta = 0$) and power for Multivariate Normal outcomes with continuous covariate. Nominal detection level (0.05) is marked with a solid black line.

A3 Simulation results: Empirical Size

Size for all simulations with normal outcomes are presented in Table 1.

Size for all simulations with Negative Binomial outcomes are presented in Table 2.

Design	Scenario	G	GO	GO.perm	BETADISPER	DTH
Balanced	S0	2	0.034	0.032	0.036	0.042
Unbalanced	S0	2	0.058	0.054	0.046	0.052
Balanced	S0	3	0.052	0.048	0.050	0.060
Unbalanced	S0	3	0.048	0.050	0.050	0.044
Balanced	S0	5	0.054	0.052	0.054	0.064
Unbalanced	S0	5	0.064	0.066	0.058	0.060
Balanced	S1	2	0.046	0.054	0.050	0.044
Unbalanced	S1	2	0.044	0.044	0.048	0.054
Balanced	S1	3	0.022	0.024	0.026	0.032
Unbalanced	S1	3	0.046	0.042	0.044	0.058
Balanced	S1	5	0.056	0.058	0.050	0.056
Unbalanced	S1	5	0.054	0.050	0.054	0.044
Balanced	S2	2	0.048	0.054	0.052	0.048
Unbalanced	S2	2	0.062	0.058	0.058	0.062
Balanced	S2	3	0.054	0.056	0.054	0.060
Unbalanced	S2	3	0.038	0.034	0.040	0.048
Balanced	S2	5	0.050	0.046	0.056	0.056
Unbalanced	S2	5	0.054	0.052	0.048	0.038
Balanced	S3	2	0.046	0.052	0.050	0.052
Unbalanced	S3	2	0.052	0.050	0.052	0.054
Balanced	S3	3	0.048	0.052	0.050	0.058
Unbalanced	S3	3	0.066	0.064	0.062	0.054
Balanced	S3	5	0.032	0.040	0.032	0.048
Unbalanced	S3	5	0.038	0.046	0.042	0.040

Table 1: Size for normal outcomes for all simulation setups

Design type	Scenario	G	GO	GO.perm	BETADISPER	DTH
Balanced	S1	2	0.068	0.068	0.062	0.068
Unbalanced	S1	2	0.046	0.042	0.048	0.044
Balanced	S1	3	0.060	0.058	0.056	0.062
Unbalanced	S1	3	0.038	0.040	0.034	0.044
Balanced	S1	5	0.044	0.046	0.046	0.058
Unbalanced	S1	5	0.068	0.068	0.064	0.062
Balanced	S2	2	0.056	0.060	0.060	0.062
Unbalanced	S2	2	0.056	0.056	0.056	0.058
Balanced	S2	3	0.044	0.044	0.050	0.042
Unbalanced	S2	3	0.058	0.062	0.060	0.056
Balanced	S2	5	0.036	0.036	0.036	0.036
Unbalanced	S2	5	0.074	0.074	0.072	0.066
Balanced	S3	2	0.042	0.044	0.040	0.046
Unbalanced	S3	2	0.050	0.050	0.048	0.038
Balanced	S3	3	0.024	0.032	0.022	0.030
Unbalanced	S3	3	0.038	0.038	0.038	0.030
Balanced	S3	5	0.042	0.052	0.038	0.062
Unbalanced	S3	5	0.030	0.032	0.030	0.026

Table 2: Size for negative binomial outcomes for all simulation setups
